# Cranial Defect Reconstruction With Custom 3D‐Printed Hydroxyapatite Scaffolds Augmented With rhBMP‐2 or Dipyridamole in a Nonhuman Primate Model

**DOI:** 10.1155/term/2466910

**Published:** 2026-01-29

**Authors:** Griffin P. Bins, Heather A. Burkart, William Molair, Samuel Kogan, Dominic A. Massary, Angel Cabrera Pereira, Adem Aksu, Frank Reinauer, Daniel A. Couture, Lukasz Witek, Christopher M. Runyan

**Affiliations:** ^1^ Division of Plastic and Reconstructive Surgery, Northwell Health, New York, New York, USA, northwell.edu; ^2^ Department of Comparative Medicine/Pathology and Department of Plastic and Reconstructive Surgery, Wake Forest School of Medicine, Winston-Salem, North Carolina, USA, wakehealth.edu; ^3^ Department of Surgery, East Carolina University, Greenville, North Carolina, USA, ecu.edu; ^4^ Department of Plastic and Reconstructive Surgery, Wake Forest School of Medicine, Winston-Salem, North Carolina, USA, wakehealth.edu; ^5^ Department of Biomedical Engineering, City College of New York, New York, New York, USA, cuny.edu; ^6^ Implants Biomaterials-Division Implants, KLS Martin SE & Co. KG, Mühlheim, Germany; ^7^ Department of Neurosurgery, Wake Forest School of Medicine, Winston-Salem, North Carolina, USA, wakehealth.edu; ^8^ Biomaterials and Regenerative Biology Division, NYU College of Dentistry, New York, New York, USA; ^9^ Hansjörg Wyss Department of Plastic Surgery, NYU Grossman School of Medicine, New York, New York, USA, med.nyu.edu; ^10^ Department of Biomedical Engineering, NYU Tandon School of Engineering, Brooklyn, New York, USA; ^11^ Department of Oral and Maxillofacial Surgery, NYU College of Dentistry, New York, New York, USA

**Keywords:** animal model, biomaterials, cranioplasty, reconstruction, tissue engineering

## Abstract

**Objective:**

Reconstruction of critical‐sized bone defects, particularly in the cranio‐maxillofacial region, presents unique challenges due to the need for integration with adjacent well‐vascularized tissue and the absence of significant load‐bearing requirements. This study evaluated the clinical readiness of bone tissue engineering (BTE) for critically sized cranial defects using custom 3D‐printed hydroxyapatite scaffolds augmented with either recombinant human bone morphogenetic protein‐2 (rhBMP‐2) or dipyridamole (DIPY) in a highly translational nonhuman primate model.

**Methods:**

Identical 5 × 5‐cm vertex guided craniotomies were created in 12 macaques: Three cynomolgus macaques served as negative controls to validate the critical size nature of the defect, while nine rhesus macaques underwent scaffold reconstruction. Subjects were divided into three groups: uncoated scaffolds (*n* = 3), scaffolds augmented with rhBMP‐2 (Infuse® Medtronic, *n* = 3), and scaffolds coated with DIPY, an adenosine A_2A_ receptor (A_2A_R) indirect agonist (*n* = 3). Bone growth and integration were assessed over 12 months through serial CT scans, followed by ex vivo micro‐CT scanning, histology, and nanoindentation testing.

**Results:**

Negative control subjects did not demonstrate new bone formation, confirming the critical defect model. Subjects treated with scaffolds through all treatment groups remained intact throughout the 12‐month follow‐up. The rhBMP‐2‐treated group exhibited bridging, ∼90% circumferentially, significantly greater than DIPY (∼9%) or the uncoated scaffold (10%) (*p* < 0.001). Bone volume within rhBMP‐2‐treated scaffolds (7621 ± 145 mm^3^) significantly exceeded that of DIPY (6466 ± 693 mm^3^, *p* = 0.03) and uncoated scaffold (6348 ± 663 mm^3^, *p* = 0.02) groups at 12 months. Quantitative histological micrograph analysis demonstrated that rhBMP‐2 scaffolds were associated with the highest bone ingrowth (∼64%) relative to DIPY (∼39%) and uncoated scaffolds (∼27%). Nanoindentation yielded superior mechanical properties (Young’s modulus and hardness) of newly generated bone with defects treated with rhBMP‐2 scaffolds (*p* < 0.05).

**Conclusions:**

Reconstructing critically sized cranial defects with custom 3D‐printed hydroxyapatite scaffolds was successful and yielded favorable results in this model. Scaffolds augmented with rhBMP‐2 demonstrated superior bone ingrowth, integration, and mechanical properties, highlighting their potential as a viable alternative to autografts and allograft materials for cranioplasty.

## 1. Introduction

Critical‐sized bone defects (CSDs) are described as bone injuries or defects that cannot spontaneously heal without intervention, presenting significant clinical and financial challenges [1]. Such defects may result from congenital anomalies, infections, high‐impact trauma or secondary to cranioplasty, and tumor extirpative surgeries [1, 2]. Autologous cranial bone flaps, while commonly utilized to repair craniotomy defects, are often associated with complications such as slow resorption, which can compromise both functional stability and cosmetic outcomes [3, 4]. In adults, bone flaps commonly develop fibrous scarring rather than successfully osseointegrating, highlighting the necessity for alternative approaches to improve long‐term outcomes [3].

Autologous bone grafts, known for their osteoconductive, osteoinductive, and osteogenic properties, are considered the gold standard for critical‐sized defect reconstruction [5, 6]. However, they are associated with respective limitations, such as donor site morbidity, limited quantity, and/or poor resorption kinetics [7, 8]. Furthermore, harvesting autologous bone can result in complications such as chronic pain, numbness, and gait disturbances, necessitating viable substitutes [6, 9]. The limited availability of autologous grafts further underscores the need to explore viable alternative solutions, such as synthetic materials [10, 11].

Various synthetic materials, such as polyether ether ketone (PEEK) and porous polyethylene (Medpor), have gained widespread adoption for cranioplasties [12, 13]. These materials offer structural integrity, cosmetic advantages, and tissue ingrowth without the risk of resorption [14]. However, they lack osteoinductive and osteoconductive properties required for direct bone integration [15]. Clinical data have also highlighted limitations in long‐term performance, particularly in pediatric or regenerative contexts. PEEK implants, for example, have been associated with complications such as infection, poor fit, and a high rate of revision surgeries, as shown in a recent 10‐year review of FDA MAUDE adverse event reports [16]. Beyond clinical complications, PEEK’s biological inertness results in suboptimal bonding with bone and soft tissue, limiting its integration and long‐term stability in orthopedic applications [17]. While osseointegration is not a priority in most adult cranioplasty cases, scenarios such as pediatric reconstructions or defects in irradiated or infected fields demand improved regenerative strategies [18]. These limitations underscore the need for osteoconductive alternatives like hydroxyapatite (HA), particularly in settings requiring durable bone integration and regeneration.

Calcium phosphate (CaPO_4_)–based ceramics, such as beta‐tricalcium‐phosphate (β‐TCP) and HA, are biocompatible, resorbable, and chemically similar to bone. These ceramics, in their bulk or particulate form, haveFDA‐approval for specific craniofacial applications and, through additive manufacturing (3D‐printing), can be customized for complex defect geometries [19, 20]. Preclinical animal models and initial clinical trials [21, 22] have successfully demonstrated porous HA scaffolds to be at least as effective as autologous bone grafts in terms of complication rates and structural outcomes [23–25]. However, a significant limitation remains: osseointegration with surrounding calvarial bone is inferior, with only ∼35% of patients achieving over 50% of radiographic union [26]. This underscores the need for further advancements to improve scaffold integration and enhance clinical outcomes. Among the various CaPO_4_ options, HA is favored over β‐TCP for large, critically sized defects due to its superior mechanical stability and slower resorption profile, which preserves structural integrity during prolonged healing [27, 28]. In contrast, β‐TCP resorbs more rapidly in vivo, which may lead to premature scaffold degradation before adequate bone formation occurs [27]. Recent studies have shown that 3D‐printed HA scaffolds with multiscale porosity not only support cell adhesion and angiogenesis but also maintain long‐term dimensional stability and promote integration with host tissue in large animal and porcine mandibular models [28–30]. These attributes, combined with HA’s close resemblance to the mineral phase of native bone, justify its selection in this study over more rapidly degrading ceramics like β‐TCP.

While substantial progress has been made with bioceramic scaffolds, the incorporation of osteogenic agents and surface coatings has emerged as an essential strategy for promoting more rapid and robust bone regeneration [31–34]. Among these agents, recombinant human bone morphogenic protein‐2 (rhBMP‐2) is one of the most extensively studied due to its strong osteoinductive potential and ability to activate Smad‐mediated transcription pathways that promote mesenchymal stem cell differentiation into osteoblasts in preclinical models [8, 35–39]. Preclinical and clinical studies, including in nonhuman primate cranial models, have demonstrated rhBMP‐2’s ability to accelerate bone regeneration and improve scaffold integration [40, 41]. However, its application in craniofacial procedures remains limited due to potential adverse effects such as heterotopic ossification (HO), inflammation, and dose‐dependent complications [41]. These concerns underscore the need for optimized delivery systems and carefully titrated dosing. Although not routinely used in cranioplasty, their potent bioactivity justifies their evaluation in complex or critical‐sized defects. To explore safer and potentially equivalent alternatives, dipyridamole (DIPY), an indirect adenosine A_2A_ receptor agonist with established vasodilatory properties [42], has been investigated for its osteogenic effects [43, 44]. DIPY has been evaluated in both small (e.g., murine) [45, 46] and large (e.g., rabbit and sheep) animal cranial models, demonstrating favorable results [45, 47]. Notably, 3D‐printed bioceramic scaffolds capable of delivering DIPY have shown effectiveness in reconstructing cranial defects without adversely affecting cranial suture patency in a skeletally immature rabbit model [45].

This study aimed to establish a clinically relevant, translational model of cranioplasty reconstruction by evaluating custom 3D‐printed HA scaffolds augmented with rhBMP‐2 or DIPY in nonhuman primates. While nonhuman primate models are ethically challenging, their close similarity to human craniofacial biology offers invaluable insights that are less achievable in other large animal models such as swine or canine. To the authors’ knowledge, this represents the first investigation of using custom 3D‐printed ceramic devices for cranioplasty procedures in nonhuman primates. The inclusion of both rhBMP‐2 and DIPY allowed us to compare two mechanistically distinct osteogenic strategies: one with well‐established efficacy but known dose‐dependent risks, and the other a promising alternative with a potentially safer profile. This design addresses the limitations of current materials and therapeutic approaches and advances regenerative strategies for complex cranial defect repair.

## 2. Methods

### 2.1. Ethical Justification and Animal Model Selection

Initially, swine models (specifically Yucatan mini‐pigs and Gottingen mini‐pigs) were selected due to their relevant size and skeletal maturation timelines. However, during preliminary experiments, significant frontal sinus pneumatization occurred, creating direct sinus communication and substantially increasing infection risks. Moreover, such sinus pneumatization prevented the creation of true critical‐sized defects, as spontaneous healing occurred (validated via serial CT scans at 4–6 months). Given these constraints, female, skeletally mature cynomolgus and rhesus macaques (*n* = 12; 3 and 9, respectively) were selected due to their analogous craniofacial anatomy, minimal sinus pneumatization, and demonstrated suitability for creating large calvarial defects. Nonhuman primates exhibit Haversian bone remodeling, human‐like bone mineral density, and analogous healing biology. Historical precedent supports the use of nonhuman primates in craniofacial bone tissue engineering (BTE) research [48, 49]. All procedures were explicitly reviewed and approved by our institutional IACUC, ensuring compliance with international ethical guidelines and adherence to the principles of Replacement, Reduction, and Refinement (3Rs).

### 2.2. Scaffold Fabrication and Preparation

Patient‐specific HA scaffolds (KLS Martin SE & Co. KG, Tuttlingen, Germany) were produced with HA slurry LithaBone™ HA 400 (Lithoz, Vienna, Austria). The CeraFab 7500 system (Lithoz, Vienna, Austria) was used to polymerize the slurry by exposure of each 25‐μm layer of the photoactive polymer to a blue LED light at a resolution of 50 μm in the *x*/*y* plane. The green body of the scaffold was then produced layer‐by‐layer [40]. After the green body was removed from the building platform of the printer with a razor blade, it was cleaned with LithaSol 20™ (Lithoz, Vienna, Austria) and pressurized air. The polymeric binder needed to keep the ceramic particles in place was decomposed during a thermal treatment regime, followed by sintering to increase the density of the ceramic particles. The sintering procedure incorporated a dwell time of 3 h between 1200°C and 1300°C to tune overall microporosity by incomplete sintering. Differences in green body shrinkage due to different maximal sintering temperatures were compensated for by adjusting (e.g., oversizing) the dimensions of the green body in all three axes so that the macro‐ and microarchitectures after sintering were identical. The sintered scaffolds were transferred onto a sterile bench, packed for incorporation into the operation workflow, and gamma sterilized. The 0.6 × 0.6 mm scaffolds were 4.0 mm thick, with pores spaced 0.9 mm apart. Each *s*caffold was custom‐designed for each subject based upon pre‐operative CT scans, where custom 3D‐printed cutting guides facilitated precise fit of the scaffolds into the surgically induced craniotomy defects.

### 2.3. DIPY and rhBMP‐2 Dosing and Loading

The dosage of DIPY needed for adequate binding to the scaffold was determined using a binding assay as described [45, 47]. Briefly, 3D‐printed cylindrical HA (Karl Leibinger Medizintechnik GmbH & Co KG; Tuttlingen, Germany) scaffolds were obtained measuring 10 mm in diameter and 5 mm in height. These scaffolds were immersed for 24 h in the dark at 37°C of three different concentrations of DIPY solution: 10, 100, and 1000 μM. After soaking, the scaffolds were washed with phosphate‐buffered solution and placed in a 24‐well plate with 1.5 mL of fresh PBS for another 24 h at 37°C in the dark. After the 24‐h soak, the scaffold was removed, the PBS solution was mixed with equal parts 100% EtOH, and the concentration of released DIPY was measured using a Tecan Infinite M200 spectrometer set to 295‐nm excitation and 485‐nm emission. The scaffold was rinsed and placed in a new 24‐well plate with fresh PBS, and the release of DIPY was measured daily for 10 days. The dose of rhBMP‐2 selected (0.2 mg/mL) aligns with previously published concentrations used effectively in preclinical bone regeneration studies involving bioceramic scaffolds. Specifically, Lopez et al. [43] demonstrated successful alveolar bone regeneration using rhBMP‐2 at 0.2 mg/mL, achieving significant bone formation comparable to DIPY‐coated scaffolds in a translational alveolar cleft model [50].

### 2.4. Surgical Procedure and Animal Groups

Upon approval from the Institutional Animal Care and Use Committee (IACUC Protocols A18‐011 and A21‐012), 12 female, skeletally mature macaques (three cynomolgus macaques and nine rhesus macaques) were acquired and underwent a quarantine period before being subjected to any study procedures. After release from quarantine, pre‐operative, baseline, CT scans were acquired after which the subjects underwent standardized craniotomies using subject‐specific cutting guides to surgically induce a 5‐cm diameter craniotomy. All subjects were randomly divided into one of the four treatment groups (Table 1).

**Table 1:** Treatment groups.

Group	Description	Subject count
No scaffold	No reconstruction performed—negative CTRL	3
Uncoated scaffold (NS)	Reconstruction with custom 3DP hydroxyapatite scaffold, without exogenous growth factors	3
rhBMP‐2 (BMP)	Reconstruction with custom 3DP hydroxyapatite scaffold, with the addition of reconstituted 4.2‐mg rhBMP‐2 (Medtronic Infuse®) to the scaffold in the OR	3
Dipyridamole (DIPY)	Reconstruction with custom 3DP hydroxyapatite scaffold, with the addition of 1000 μM dipyridamole to the scaffold in the OR	3

Following the acclimation period and pre‐operative CT imaging, animals were sedated with ketamine (15 mg/kg IM) and given atropine (0.04 mg/kg IM) and buprenorphine (0.02–0.04 mg/kg IM). Animals were intubated and maintained on isoflurane (0%–5%) or sevoflurane (0%–8%) inhalant anesthesia. The top of the head was clipped, prepped with chlorhexidine and alcohol, and draped for aseptic surgery. Cefazolin (25 mg/kg IV) was given pre‐operatively and repeated every 90–120 min during surgery. Bupivacaine (up to 2 mg/kg SC) with epinephrine was injected at the surgical site prior to incision. A midline sagittal incision was made along the length of the cranium through the skin and subcutaneous tissue. The temporal muscles and periosteum were incised, elevated, and retracted laterally to expose the skull. Subsequently, using custom 3D‐printed cutting guides per subject, a craniectomy was performed to surgically induce a 5 cm in diameter calvarial defect. Incidental durotomies were repaired with 4‐0 Nurolon suture when able or left to heal secondarily. Hemostasis was controlled with pressure, cautery, gel foam, and bone wax. The experimental groups received a custom 3D‐printed scaffold, each secured using a single 1 mm poly‐D‐di‐lactic acid (PDDLA, Sonic Weld) plate. The plates were heated to allow for adaptation to the curvature of the scaffold and surrounding bone and held to the mid‐portion of the scaffold with a 4‐0 Vicryl suture and a single, 4‐mm Sonic Weld screw to the native bone on either side of the implant. The incision was closed routinely in layers (temporal muscles, subcutaneous tissues, and skin). Postoperatively, animals were given ceftiofur sodium (2.2 mg/kg IM), buprenorphine (0.02 mg/kg IM), NSAIDs (ketoprofen 5 mg/kg IM/SC or meloxicam 0.1–0.2 mg/kg PO), and famotidine (0.5 mg/kg IM). Scaffold fit within guided craniotomies was deemed to be excellent; although the area of direct contact was noncircumferential, all subjects had some degree of direct scaffold‐to‐bone contact.

### 2.5. Longitudinal CT Imaging and Bridging Assessment

Following the surgical procedure, serial CT scans were obtained every 2 months to assess the progression of scaffold integration. Pre‐implantation, baseline CT was also acquired. Each scan was imported into Materialise Mimics® (Build 14.0.0.3632) for 3D reconstruction using bone density thresholds (225–3070 HU). Scaffold incorporation was quantified by placing landmarks in a “clock face” pattern around the circular implant, dividing the perimeter into 12 equal segments. Percent bridging within each segment was independently scored by two reviewers and averaged to assess integration over time. At 12 months postimplantation, final CT imaging was performed, after which subjects were euthanized under deep sedation from pentobarbital IV, followed by systemic flushing with 0.9% NaCl or lactated Ringer’s solution and fixation with 10% neutral buffered formalin (NBF), per IACUC protocol. Heads (inclusive of skull, brain, and overlying soft tissues) were collected intact and placed in 10% NBF for further fixation. Postmortem cranial samples including underlying dura and overlying periosteum/temporalis muscle were subjected to micro‐CT imaging and then bisected for histology and nanoindentation testing.

### 2.6. CT Imaging

Postmortem, high‐resolution computed tomography (CT) imaging was performed using a GE Discovery MI DR PET/CT (GE HealthCare, Chicago, IL) to assess scaffold volume and integration with the surrounding native bone. Scans were acquired at a slice resolution of 18 μm, with an x‐ray energy of 120 kVp, a current of 175 mA, and a DFOV of 22 cm. The resulting volumetric datasets were imported into Materialise Mimics® for segmentation using identical threshold parameters as the standard CT images (225–3070 HU). The volume (mm^3^) of ossified tissue inclusive of scaffold and new bone growth (but excluding surrounding, native bone) was measured for each explant. All implanted scaffolds had identical diameters and pore sizing and spacing.

Peripheral scaffold integration was further assessed by segmenting the interface between the implant and native bone. The implants were denoted at eight evenly distributed locations around the circular implant’s perimeter. The interfaces with native bone were visualized using sagittal and coronal slices, and the percent of the area between the native bone and implant with bridging was assessed by two operators and then reported as an average.

### 2.7. Histological Analysis

After μCT scanning, the specimens were gradually dehydrated in a series of alcohol solutions ranging from 70% to 100% ethanol. Following the dehydration process, the samples were embedded in a methacrylate‐based resin (Technovit 9100, Heraeus Kulzer GmbH, Wehrheim, Germany), according to the manufacturer’s instructions. The blocks were then cut into slices (∼300 μm thick) centering the region of interest corresponding to its long axis, in a mesial distal direction within the defect, with a precision diamond saw (Isomet 2000, Buehler Ltd., Lake Bluff, IL) glued to acrylic plates with an acrylate‐based cement (Technovit 7210 VLC, Heraeus Kulzer GmbH, Wehrheim, Germany) and allowed to set for 24 h prior to grinding and polishing. The sections were reduced to a final thickness of ∼80 μm by means of a series of silicon carbide (SiC) abrasive papers (400, 600, 800, and 1200; Buehler Ltd., Lake Bluff, IL) utilizing a grinding/polishing machine (Metaserv 3000, Buehler Ltd., Lake Bluff, USA) under irrigation. Subsequently, the samples were stained with Stevenel’s blue and van Gieson’s picro fuchsin (SVG). Stevenel’s blue stains cells and extracellular structures in a subtle gradation of blue tones. The counterstain, van Gieson’s picro fuchsin, stains collagen fibers green or green–blue; bone, orange or purple; osteoid, yellow–green; and muscle fibers, blue to blue–green. Qualitative histologic analysis was performed by a blinded veterinary pathologist.

Utilizing the OpenCV library for Python 3, a program was designed to analyze histological slides. The primary outputs for this program were percent bony ingrowth into the scaffold at the internal and external surfaces, around the entire periphery, and within the scaffold itself. Color gates in hue, saturation, value (HSV) colors were assigned to scaffold and bone. Scaffold was assigned gates of [0, 0, 0] to [70, 67, 90], and bone was assigned gates of [155, 57, 67] and [179, 202, 187]. A simple graphic user interface (GUI) was designed to allow the user to pick an appropriate kernel size to define the outer border of the scaffold. We then used a centroid calculation to define the center of the scaffold and found the extreme points of the scaffold. From here, bone in the center, sides, circumference, and above and below centerline was derived and reported using the previously established gates. Finally, an image was generated for the user to check and ensure that the calculations were performed using the desired points.

### 2.8. Mechanical Property Testing

Histological slides were subjected to nanoindentation analysis using a nanoindenter (Hysitron TI 950, Minneapolis, MN) equipped with a Berkovich three‐sided, diamond pyramid probe. Nine indents per specimen were performed using a previously developed loading profile with a loading rate of 60 μN/s for 5 s followed by a constant peak load of 300 μN, termed as holding time, for 10 s and subsequent unloading in 2 s to produce load–displacement curves corresponding to each area indented [51, 52]. This nanoindentation testing was performed as a comparative analysis of mechanical strength (Young’s modulus) and hardness of bone between areas within the lattice‐like nature of the scaffold and native bone present around the induced defect site.

Slides were first imaged followed by a scratch test to confirm whether the samples were flat with an error in the indenter’s axial displacement recorded to be < 1 μm over a scan area of 50 μm^2^. Regions were further assessed for the presence of bone in the testing site following which nine indentations were performed in a sequential manner, with each indent separated by 10 μm from each other. Tests were performed at room temperature with appropriate passive and active noise isolation and damping. The set of nine load–displacement curves were fitted using a standard model using the Hysitron TriboScan quasistatic data analysis package to compute reduced elastic modulus, *E*_*r*_ (GPa) (Equation (1)), and hardness, *H* (GPa) (Equation (2)), of bone tissue [28].

(1) Er=π2AhcS,

(2) H=PmaxAhc.

In these equations, *S* is the stiffness, *h*_*c*_ is the contact depth, *P*_max_ is the maximum force, and *A*(*h*_*c*_) is the contact area computed from the Hysitron TriboScan software. Elastic modulus of the bone, *E*_*b*_, was then evaluated from the obtained reduced modulus, *E*_*r*_, using the Hertzian contact mechanics (Equation (3)) relationship.

(3) Eb=1−vb2EiErEi−1−vi2Er,

where *v*_*b*_ is the Poisson ratio of the bone and *E*_*i*_ and *v*_*i*_ are the elastic modulus and Poisson ratio of the indenter, respectively.

### 2.9. Statistical Analysis

All data were analyzed using insert software, SPSS v29 (IBM Corp., Armonk, NY). The results are presented as mean ± standard deviation (SD) unless otherwise stated. Normality of data distribution was assessed using the Shapiro–Wilk test. For normally distributed data, comparisons between two groups were performed using the unpaired Student′s *t* test, while comparisons among more than two groups were analyzed using one‐way ANOVA followed by Tukey’s post hoc test. For non‐normally distributed data, the Mann–Whitney *U* test or Kruskal–Wallis test with Dunn’s post hoc correction was applied. A *p* value of less than 0.05 was considered statistically significant.

## 3. Results

### 3.1. DIPY Release

The binding assay results indicated concentration‐dependent binding and release kinetics of DIPY from the HA scaffolds over the 10‐day observation period (Figure 1). Scaffolds incubated with 1000 μM DIPY displayed the highest initial binding (Figure 1(c)), with a gradual sustained release, maintaining detectable DIPY concentrations (∼10 μM) through Day 10. Scaffolds treated with 100 μM DIPY (Figure 1(b)) demonstrated moderate initial binding and a progressive release, reaching undetectable levels by Day 6. The lowest concentration tested (10 μM DIPY; Figure 1(a)) showed minimal binding, rapidly declining to undetectable levels by Day 3. Based on these results, the 1000 μM DIPY concentration was selected for the subsequent scaffold preparation due to its higher initial binding efficiency and prolonged release profile.

Figure 1DIPY binding and release from hydroxyapatite scaffolds. DIPY release was concentration‐dependent ((a) 10 μM, (b) 100 μM, and (c) 1000 μM), with sustained release through Day 10 in the (c)1000‐μM group, moderate release in the (b) 100‐μM group (undetectable by day 6), and minimal binding in the (a) 10‐μM group (undetectable by Day 3). A sample size of *n* = 3 was performed in triplicate with data presented as mean and standard deviation.

**Figure:**
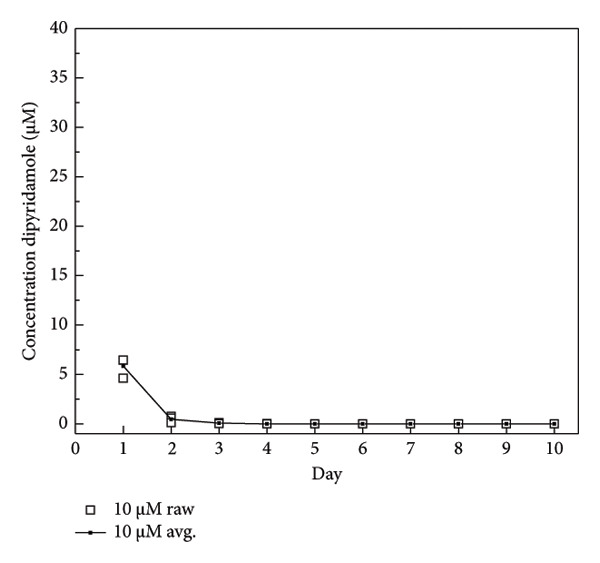
(a)

**Figure:**
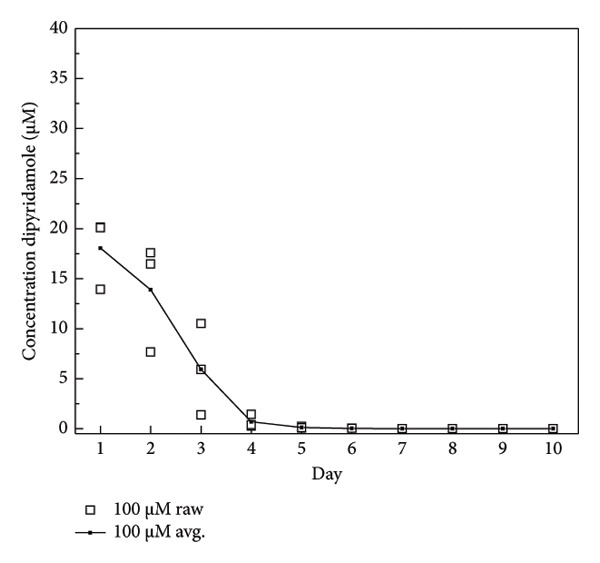
(b)

**Figure:**
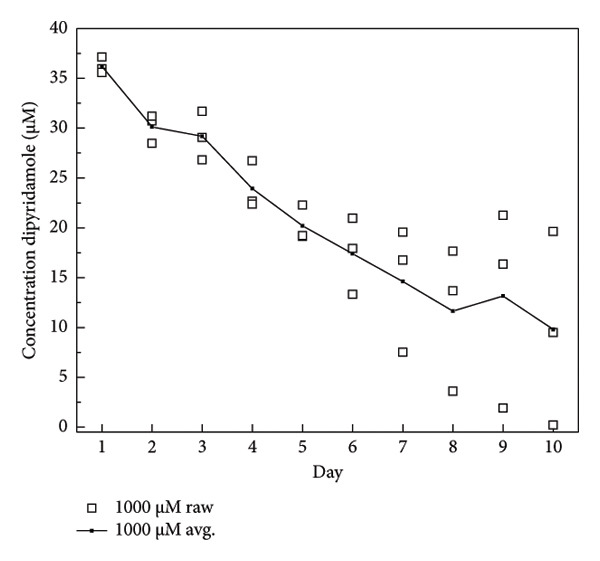
(c)

### 3.2. Clinical Evaluation

**Figure 2:**
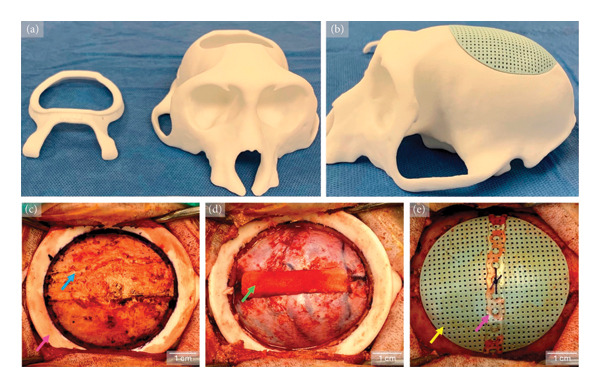
Subject‐specific cutting guides and scaffolds. Pre‐operative CT scans were used to design each subject’s (a) 3D‐printed cutting guide and (b) corresponding hydroxyapatite (HA) scaffold. Both were fabricated with a 5 cm diameter to match the superior aspect of the cranium. Representative intraoperative images show (c) cutting guide (pink arrow) positioned after sagittal incision with exposed calvarium (blue arrow); (d) post‐craniectomy with gel foam applied to address durotomies (green arrow); and (e) custom HA scaffold (yellow arrow) implanted and secured with a PDDLA plate (purple arrow).

Throughout the 1‐year postoperative period, there were no incidences of infection, graft loss, or wound breakdown (Figure 2). One subject in the no‐scaffold group experienced self‐limited seizures immediately after surgery and 4 months after surgery. No treatments or medical management were required. Another subject, from the DIPY group, experienced surgical complications of a subdural hematoma in the left hemisphere and brain tissue herniation through iatrogenic durotomy in the right hemisphere that required limited excision. Postoperatively, the animal experienced mild hemiparesis of the left side and abnormal clinical signs, which gradually improved and/or was resolved over 3–4 weeks.

### 3.3. Imaging Analysis

**Figure 3:**
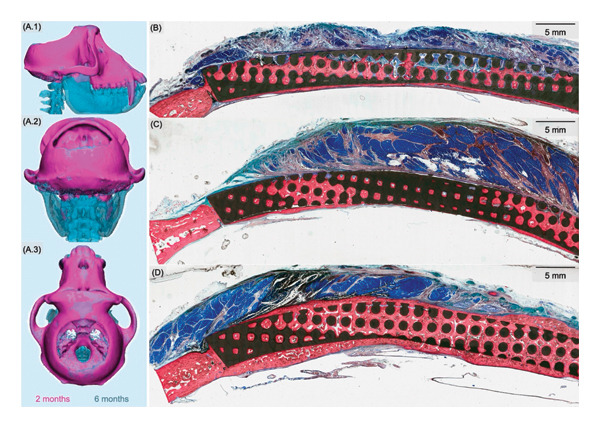
Volumetric and histologic analysis. (A.1, A.2, A.3) Three subjects underwent craniectomy without cranioplasty and underwent CT every 2 months following surgery. At their 6‐month CT, no significant bone growth was observed, and the 5‐cm defects were deemed to be critical. Representative histological micrographs showing soft tissue (blue), mineralized tissue (red), and scaffold (black) were analyzed for (B) NS, (C) DIPY, and (D) BMP samples. Both blinded pathologist assessment and automated viewing software identified greater bone growth in BMP samples. Specifically, bone growth occurred to a greater extent on deeper aspects more proximal to the dura.

**Figure 4:**
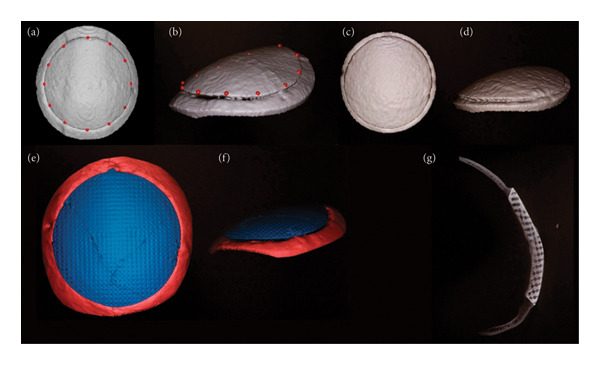
Longitudinal CT and postmortem CT analysis. By applying thresholds for bone (250–3070 HU), 3D models of the cranioplasty and surrounding bone were created. (a) Marking models in a clockwise pattern divided the cranioplasty’s perimeter into 12 distinct regions. Two independent raters scored these regions for the degree of bridging. (b) Bridging percentage was described as the presence of approximation between the cranioplasty and native bone. (c and d) While the final longitudinal CT obtained 12 months postoperative was used to measure the presence of approximation, (e and f) micro‐CT created a higher resolution model, which could assess integration. (e) After obtaining micro‐CT, a single instance of subclinical implant fracture was observed. Following fracture, the implant remained in place and the subject showed no clinical signs of distress. These high‐resolution models were able to evaluate bony ingrowth within the implant by measuring differences in volume. (g) Mid‐sagittal slice of a BMP sample demonstrating bony union/integration with adjacent skull. To assess the degree of integration, equally spaced landmarks were placed on the 3D reconstructions around the periphery of the interface (see Table 2).

Subjects without reconstruction following craniectomy (*n* = 3) showed no evidence of bone growth on serial CT scans at 6 months, validating the model/size as a critical defect (Figure 3(A.1, A.2, A.3)). In the remaining nine subjects, new bone growth into the scaffold was evaluated by scoring circumferential bridging on bimonthly CT scans. Gross circumferential bridging (100%) was observed consistently in the scaffolds augmented with rhBMP‐2 at all the time points (Figure 4), whereas DIPY‐ and untreated scaffolds exhibited progressive bridging over time (Table 2). However, due to the sample size and variation, differences between groups were not significant. To differentiate new bone from the HA scaffold in CT analysis, we relied on distinct radiodensity values: The scaffold exhibited uniform, high radiopacity, whereas newly formed bone appeared less radiodense and more heterogeneous, particularly at the bone–scaffold interface. These differences allowed for visual and software‐aided discrimination of the scaffold material versus new bone on both standard and high‐resolution scans.

**Table 2:** Demographics and imaging results.

Demographics			Standard CT bridging results						Micro‐CT results	
Subject	Sex	Age at TOS (yr)	2 months	4 months	6 months	8 months	10 months	12 months	Bridging	Volume (mm^3^)
NS1	Female	15	55%	73%	74%	73%	76%	79%	0%	5793
NS2	Female	12	54%	94%	96%	98%	97%	100%	5%	6289
NS3	Female	9	86%	95%	100%	100%	100%	100%	24%	6961
BMP1	Female	13	n/a	100%	100%	100%	100%	100%	100%	7514
BMP2	Female	17	100%	100%	100%	100%	100%	100%	90%	7586
BMP3	Female	9	100%	100%	100%	100%	100%	100%	81%	7763
Dipy1	Female	17	85%	93%	95%	98%	98%	98%	2%	5936
Dipy2	Female	12	68%	69%	n/a	79%	81%	86%	4%	6325
Dipy3	Female	9	79%	83%	92%	91%	93%	93%	22%	7137
NS Avg		**12**	**65%**	**87%**	**90%**	**90%**	**91%**	**93%**	**10%**	**6348**
BMP Avg		**13**	**100%**	**100%**	**100%**	**100%**	**100%**	**100%**	**90%**	**7621**
Dipy Avg		**13**	**77%**	**82%**	**94%**	**89%**	**91%**	**93%**	**9%**	**6466**
*p* value (ANOVA)			**0.073**	**0.158**	**0.450**	**0.414**	**0.404**	**0.462**	**< 0.001**	**0.036**

*Note:* They are the average, just a illustrative denotation to differ from the individual values.

Abbreviation: TOS, time of surgery.

Micro‐CT analysis offered enhanced spatial resolution, enabling a more precise quantification of scaffold integration and bone bridging. While standard CT often overestimated the extent of bone–scaffold contact, micro‐CT revealed that intimate apposition between native bone and scaffold was less extensive than initially appreciated. Quantitatively, the rhBMP‐2, DIPY, and no‐scaffold groups had average bridging of 90%, 9%, and 10%, respectively (*p* < 0.001, Table 2). Correlation between the two independent raters was high with an ICC = 0.998 and 0.995 on longitudinal and micro‐CT, respectively. Micro‐CT analysis of harvested scaffolds further demonstrated a significantly greater volume of bone formation in the rhBMP‐2‐treated scaffolds (7621 ± 145 mm^3^) compared to the naked, uncoated, scaffolds (6348 ± 663 mm^3^, *p* < 0.021) but not with the DIPY‐augmented scaffolds (6466 ± 693 mm^3^, *p* < 0.033) after Bonferroni correction. HO was observed within the overlying muscle in two of the three rhBMP‐2 subjects. No such findings were observed in other groups. All volumetric calculations excluded HO.

### 3.4. Histologic Evaluation

Qualitative histologic analysis included both total and regional bony ingrowth. Bony ingrowth was observed in all treatment groups (Figures 3(b), 3(c), 3(d)). The rhBMP‐2 group demonstrated the highest degrees of bony ingrowth within both the scaffold pores and at the periphery relative to the other groups; however, there were no significant statistical differences (Table 3). The rhBMP‐2 group presented with increased incorporation with ∼65% of the porous aspects of the scaffold being filled with bone (DIPY, 39%; NS, 27%) and increased circumferential growth with approximately 58% of the scaffold’s periphery being surrounded by bone (DIPY, 22%; NS, 18%). This new growth occurred to a greater extent on the deep surface of the scaffold adjacent to the dura, compared to the superficial surface (66% vs. 48%, respectively). Similar increased bone formation at the deep surfaces was also observed with the DIPY (28% vs. 11%) and NS (25% vs. 3%) groups.

**Table 3:** Histologic evaluation.

Subject	Scaffold interior	Superficial	Deep
BMP1	49%	5%	58%
BMP2	79%	81%	76%
BMP3	68%	59%	66%
Di1	13%	0%	1%
Di2	31%	20%	42%
Di3	74%	13%	42%
NS1	9%	0%	2%
NS2	17%	8%	13%
NS3	55%	3%	59%

Group average			

BMP	65%	48%	66%
Di	39%	11%	28%
NS	27%	3%	25%

*p* value (ANOVA)	0.226	0.114	0.120

Blinded qualitative histologic assessment identified mononuclear and multinucleated cells to variably line the scaffold material, with associated resorption of the scaffold. Mesenchyme and new bone replaced the scaffold material through membranous ossification. The new bone was composed of woven bone with variable degrees of bone remodeling characterized by osteocytes within lacunae, cement lines, and some osteon/lamellar bone formation. Osteoblasts regularly line the new bone, and the osteocytes are present within lacunae and are variably organized. Randomly, osteoclasts were identified within Howship lacunae. Overall, new bone formation within and around the scaffold was greater in the rhBMP‐2 group when compared to DIPY and NS. Due to the individual variation within each group, a treatment effect could not be determined between the NS and DIPY groups.

### 3.5. Strength Testing

Young’s modulus was determined to be greater in the rhBMP‐2 group (13.384 ± 0.45 GPa) than native bone (11.187 ± 0.35 GPa), NS (9.707 ± 0.42 GPa), and DIPY (5.204 ± 0.43 GPa) groups (Figure 5). Hardness was found to be equivalent between rhBMP‐2 (0.464 ± 0.02 GPa) and native bone (0.444 ± 0.01 GPa), and both were found to be significantly different relative to NS (0.358 ± 0.02 GPa) and DIPY (0.244 ± 0.02 GPa). All other findings regarding Young’s modulus and hardness were statistically significant (*p* < 0.05).

Figure 5Mechanical strength testing. Elasticity (a) and hardness (b) were obtained via nanoindentation testing. Bone growth in rhBMP‐2 samples was found to be as hard as native bone and more resistant to deformation. Bone growth in dipyridamole and untreated samples did not reach the strength of native bone and similarly were not found to be as hard or resistant to deformation as the bone growth in rhBMP‐2 samples. While other analyses found no difference between dipyridamole and untreated subjects, dipyridamole samples were notably found to have inferior bone growth. The horizontal dashed red line denotes the mean with corresponding 95% CI shown by the black horizontal lines. Each group had one slide tested per subject; thus, *n* = 3 per group. Each set of indents had nine measurements, which were subjected to statistical analysis.

**Figure:**
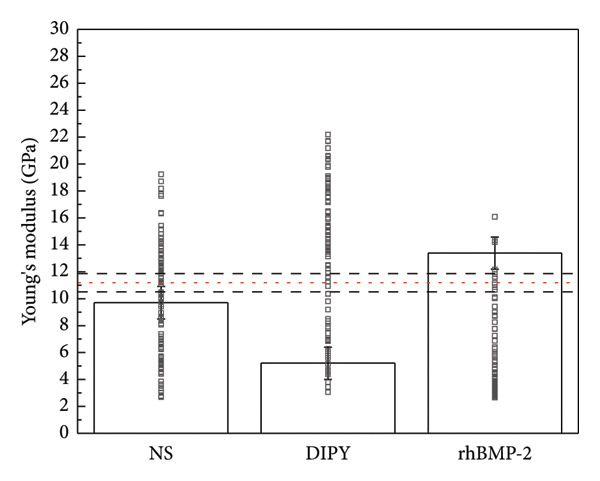
(a)

**Figure:**
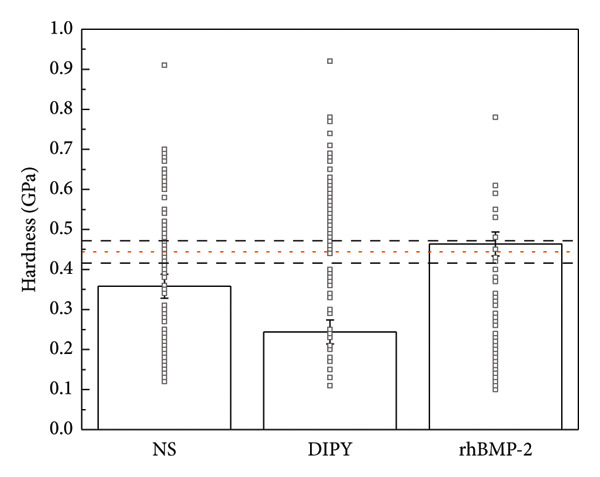
(b)

## 4. Discussion

Reconstruction of critical calvarial defects remains a complex clinical challenge, traditionally treated using autologous bone grafts or alloplastic substitutes. Commonly utilized alloplastic materials, such as polymethyl methacrylate, poly‐etheretherketone, and titanium mesh, provide structural stability and ease of use but are associated with risks of wound healing complications, potential for infection, and in certain cases extrusion. These risks, however, are not unique to synthetic materials and are shared across various cranioplasty techniques, including autologous reconstruction [53–56]. Autologous cranioplasty with the patient’s own craniotomy bone flap remains a standard approach, preferred for its biological compatibility and ability to restore original curvature, whereas autogenous bone graft from calvarium, rib, or iliac crest is widely regarded as the gold standard for reconstruction. Unfortunately, these grafting sites are associated with significant donor site morbidity, the potential for graft resorption, and increased postoperative recovery [57].

Ceramic scaffolds, such as HA and β‐TCP, offer promising alternatives due to their biocompatibility and osteoconductivity. These materials have demonstrated the ability to integrate with surrounding bone; however, their application in large, critically sized defects is limited by their lack of osteoinductivity, which is essential for full integration [51] [58]. While both HA and β‐TCP share biocompatible and osteoconductive properties [52, 53, 59, 60], they differ significantly in their rates of absorption [52, 54–56] [60–63], where HA exhibits a slow absorption rate of less than ∼1% per year in vivo, whereas β‐TCP has a much higher absorption rate of approximately 18% per year [61]. For this study, HA was selected specifically for its slower absorption rate, ensuring long‐term scaffold integrity in the reconstruction of critically large defects.

Osteogenic molecules, such as rhBMP‐2 and DIPY, have been extensively studied for their potential to enhance scaffold integration. A series of primate studies have evaluated the use of scaffolding in conjunction with rhBMP‐2 for the treatment of calvarial defects [57–60] [64–67]. However, these studies included small defects (< 25 mm diameter) with short‐term follow‐up (< 3 months). Despite its effectiveness, rhBMP‐2 has been linked to swelling [61, 68], HO [69], and craniosynostosis when used in skeletally immature, preclinical animal models [70].

Notably, HO was observed in two of the three BMP‐2‐treated subjects in this study. HO represents a significant clinical concern, as unintended bone formation within surrounding soft tissues can complicate revision procedures, potentially impair function, and increase morbidity. These findings highlight the importance of carefully considering dosing, scaffold design, and application techniques when translating rhBMP‐2–based therapies into clinical practice. Further investigation into minimizing HO while preserving the osteoinductive efficacy of rhBMP‐2 is essential.

In contrast, DIPY increases osteogenic differentiation by preventing adenosine reuptake [38, 71]. This leads to dose‐dependent decreases in bone resorption and increased bone growth that may be comparable to rhBMP‐2 [46, 47, 71–73]. Previous studies in rabbit and sheep models have suggested equivalence between rhBMP‐2 and DIPY in promoting ingrowth into ceramic scaffolds, prompting further investigation into their comparative efficacy [47, 52].

This study focused on defects measuring 5 cm in diameter, significantly larger than those in previous studies and more comparable to critical cranial defects requiring reconstruction in humans, particularly given their curvature [26, 46, 53, 73]. To closely mimic clinical practice, custom cutting guides were designed using CAD software to ensure an optimal fit with the defect. After standardizing model geometry, including cross‐sectional area, it was established that bone within DIPY‐treated scaffolds was significantly inferior in both hardness and elastic modulus relative to control and rhBMP‐2 groups. Bone in the rhBMP‐2‐treated scaffolds demonstrated significantly greater hardness and elastic modulus relative to both to DIPY‐treated and control groups. Mechanically, bone from rhBMP‐2–augmented scaffolds was significantly more resistant to axial deformation and significantly more difficult to penetrate the surface than either DIPY‐treated or control scaffolds, and its properties were equivalent to native bone. However, this strength testing was performed only at the study’s conclusion, making it impossible to determine the exact time point at which the implant achieved native bone strength.

While increased osteogenesis was not observed in the DIPY‐treated group, the findings do not invalidate DIPY as an osteogenic adjunct to ceramic scaffold incorporation. Previous studies have demonstrated strong osteoinductive effects of DIPY when combined with collagen‐treated β‐TCP scaffolds [26, 46, 53, 55]. Impaired binding of DIPY in this study may explain the observed difference in bone growth. Interestingly, despite no significant differences in histologic bone volume between DIPY and control groups (NS), DIPY‐treated scaffolds showed significantly lower stiffness and hardness compared to NS. This unexpected result suggests that DIPY’s bioactivity may influence the bone’s biomechanical properties independently of overall bone quantity, possibly due to altered bone matrix quality or maturation. Alternatively, this could reflect the importance of substrate and fabrication modality in mediating DIPY efficacy. We have previously shown that DIPY‐augmented β‐TCP scaffolds fabricated via direct‐inkjet writing (DIW) yielded regenerated bone with elastic moduli comparable to native bone [50, 74–77], highlighting that scaffold composition and printing technique critically influence DIPY retention and downstream mechanical outcomes. In contrast, the current study used HA scaffolds fabricated by lithography‐based ceramic manufacturing (LCM), a form of vat photopolymerization, which may limit DIPY bioavailability due to differences in surface chemistry, microporosity, or drug diffusion characteristics. These findings underscore the need to tailor scaffold–drug combinations specifically for each material and fabrication method to optimize regenerative performance. Effective concentrations of > 1 μM are required to induce bone growth, and it is possible that levels were not sustained long in this larger, more translational, preclinical model. Future studies exploring scaffolds with a β‐TCP‐component may help clarify this difference. For example, a β‐TCP/HA mixture could further optimize the rate of scaffold resorption and replacement with native bone [78].

While this study validated the utility of custom 3D‐printed HA scaffolds for reconstructing critical‐sized defects in a nonhuman primate model, several limitations must be acknowledged. The small sample size and focus on a single ceramic material and dosing regimens limited the scope of findings. Additionally, the relevance of immediate cranioplasty to clinical practice may be questioned, as many cranioplasty cases involve delayed reconstruction due to elevated intracranial pressure. This timing may influence osseointegration, particularly in rhBMP‐2 scaffolds, warranting further investigation. Additionally, one untreated scaffold experienced a nondisplaced fracture, highlighting the brittleness of ceramics as a potential limitation to clinical translation. Notably, this fracture was subclinical, as the subject exhibited no signs of increased distress, pain, or impairment, and CT scans appeared normal through the 6‐month mark. The fracture was first appreciated upon evaluation of the postmortem micro‐CT, at which time previous CTs were carefully reviewed identifying the time of fracture as having occurred between 6 and 8 months postoperatively.

Although BMP‐treated scaffolds demonstrated clear advantages in terms of long‐term strength and biocompatibility, comprehensive cost‐benefit analyses are critical to justify their clinical use, particularly when compared to less expensive and widely accepted materials like PEEK or Medpor. Nevertheless, despite the small cohort sizes, all scaffold‐treatment groups in this study safely and effectively reconstructed a critically sized calvarial defect. Future investigations into optimizing ceramic composition and rhBMP‐2 dosing will be essential to facilitate the clinical translation of these promising findings.

Future research should focus on refining scaffold composition to enhance osteogenesis while minimizing brittleness and optimizing cost‐effectiveness. Exploring hybrid ceramic scaffolds and alternative bioactive molecules may bridge the gap between performance and affordability. Additionally, investigating the timing of cranioplasty and its effects on osseointegration is critical for aligning preclinical models with clinical realities. Nonhuman primate models remain valuable for translational research; however, efforts to minimize their use and focus on alternative large animal models, such as pigs, should align with regulatory and ethical standards.

## 5. Conclusion

Reconstruction of critical cranial defects was successfully achieved using large, custom 3D‐printed HA cranioplasty implants, with a 1‐year follow‐up confirming safety and efficacy across all treatment groups. Scaffolds pretreated with the osteoinductive factor rhBMP‐2 demonstrated significantly enhanced bony ingrowth and bridging, achieving complete and rapid fusion with the surrounding bone and exhibiting the most robust bone formation within scaffold interstices. Additionally, bone from rhBMP‐2–augmented scaffolds displayed mechanical properties equivalent to native bone, emphasizing its potential as a superior alternative for critical cranial defect reconstruction. This study demonstrates successful incorporation of large HA cranial scaffolds and suggests an alternative approach to alloplastic materials for surgical cranioplasty. Despite prior evidence to the contrary, the addition of DIPY did not demonstrate osteoinductivity in this model. Additionally, efforts to align preclinical models with clinical realities, including delayed cranioplasty scenarios and the use of alternative large animal models, will be critical to advancing the application of these findings.

## Conflicts of Interest

The authors declare no conflicts of interest.

## Author Contributions

Griffin P. Bins and Heather A. Burkart contributed equally to the writing of this manuscript.

## Funding

No funding was received for this manuscript.

## Data Availability

The data that support the findings of this study are available from the corresponding author upon reasonable request.
